# On the Northward Expansion of Scallops (*Pecten maximus*) Along the Norwegian Coastline

**DOI:** 10.1002/ece3.71460

**Published:** 2025-05-28

**Authors:** Ingrid A. Johnsen, Ellen Sofie Grefsrud

**Affiliations:** ^1^ Institute of Marine Research Bergen Norway; ^2^ The Norwegian Directorate of Fisheries Bergen Norway

**Keywords:** climate change, distribution, environment, *Pecten maximus*, polar shift

## Abstract

The Norwegian coastline spans from 58° N to 71° N and exhibits an environmental gradient with decreasing temperatures from south to north. Historically, the distribution of the great scallop ( *Pecten maximus* ) along the Norwegian coastline has been from Skagerrak in the south to Bodø (67° N) in the north. The northernmost distribution was documented in 2001 by scientific diving. Since 2011, monitoring has revealed a northward shift, where small populations of *P. maximus* have established themselves beyond the previous distribution boundaries along the coastline and in the mid‐western part of the Lofoten Islands at 68° N. The northward expansion of the great scallops' distribution is believed to be limited by low temperatures. However, over the past 15 years, coastal water temperatures have increased by approximately 1°C. Although a 1° temperature increase may seem modest, it reduced the time during winter with temperatures below 4°C from three to one month at the coastal station Skrova at 68° N. As the great scallop's preference for warm water is widely documented, and the timing of the northward shift seems to occur at the same time as the warmer waters are observed, we believe that the temperature increase is the main explanation for the observed northward shift in established populations of *P. maximus* .

## Graphical abstract

Since the mid‐2000s, monitoring has revealed a northward shift, where small populations of the great scallop *Pecten maximus* have established themselves beyond the previous distribution boundaries in Norway. The timing of the northward shift seems to occur at the same time as the warmer waters are observed, we believe that the temperature increase is the main explanation for the observed northward shift on established populations of *P. maximus* .

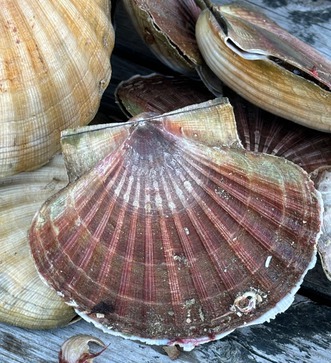

## 1 Introduction

Over the past few decades, there has been a noticeable change in both the temperature and salinity of coastal waters along the Norwegian coastline that stretches over 21,000 km from 57° to 71° north (Central Intelligence Agency 2011). Previous studies clearly indicated that the 1990s marked a transition period from the climate conditions observed between 1940 and 1990 to the period after 2000, particularly in the coastal waters from southern Norway to Lofoten at approximately 68° north (Sætre and Aure 2007; Aure et al. 2007; Albretsen et al. 2012; Aksnes et al. 2019; Johnsen et al. 2024). Approximately 25% of the increase in Norwegian coastal water temperature has been attributed to fluctuations in the North Atlantic Oscillation, whereas 75% of the warming appears to be associated with large‐scale hemispheric warming (Albretsen et al. 2012).

**FIGURE 1:**
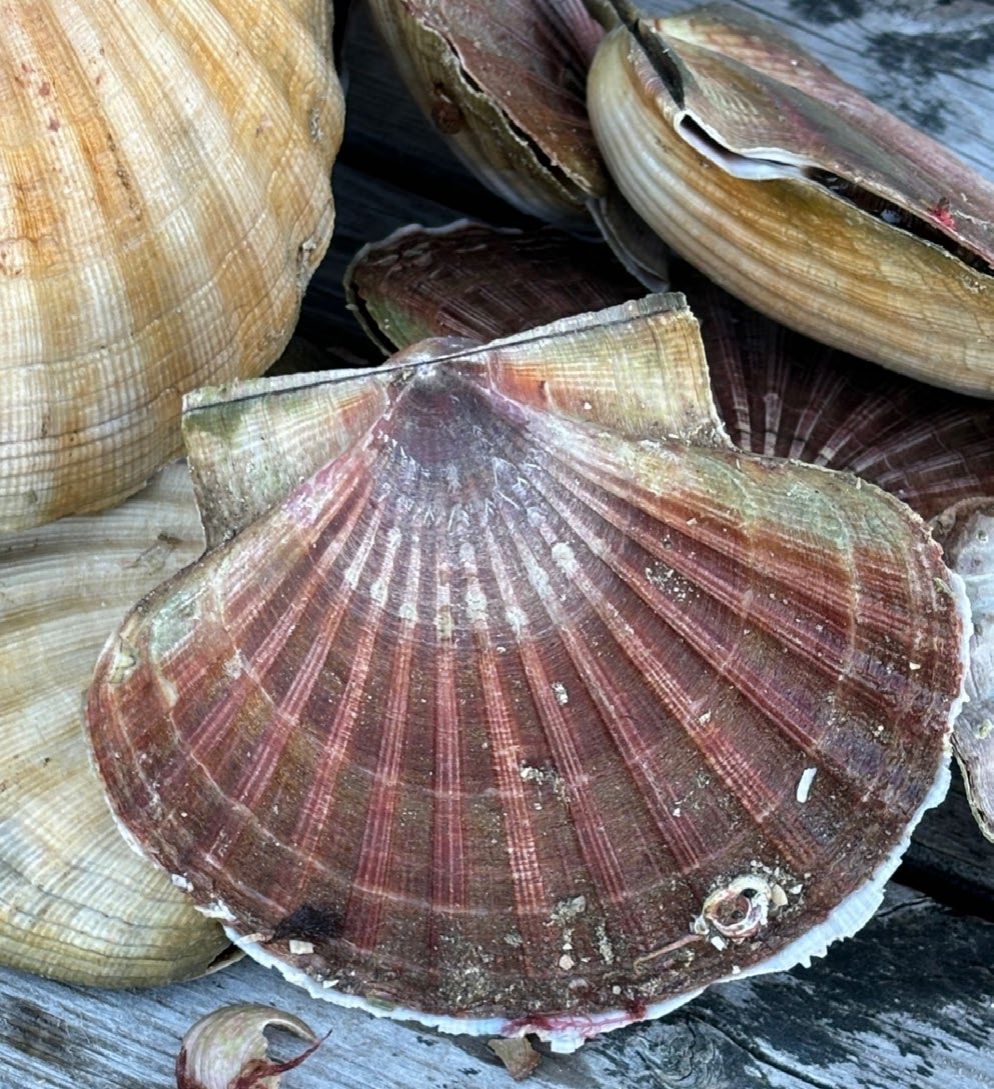
The great scallop *Pecten maximus* . Photo: E. Grefsrud.

The warming trend observed in coastal waters over the past 30 years, along with the latitudinal temperature gradient and the northward flow of a current along the Norwegian coastline, are factors that contribute to the northward dispersion of warm‐temperate species. This shift in distribution has been documented for various marine species in previous studies (Brattegard 2011; Hollowed et al. 2013; Kortsch et al. 2015; Fossheim et al. 2015; Kjesbu et al. 2022). One example, the edible crab ( *Cancer pagurus* ), was suggested to have a northerly distribution border just north of 68° N but is now commercially harvested as far north as 69° N (Bakke et al. 2019) and has been observed in waters close to 71° N (Brattegard 2011). The great scallop ( *Pecten maximus* ) is distributed from the Iberian Peninsula in the south to Vesterålen in northern Norway (Wiborg and Bøhle 1974; Duncan et al. 2016) (Figure 1). Along the Norwegian coast, the great scallop ( *P. maximus* ) is abundant in the range of 63°–66° N, with the highest abundances observed at 64° N (Strand and Parsons 2006); the same area where a commercial diver‐fishery was established in the early 1990s. According to the yearly landing data provided by The Norwegian Fishermen's Sales Organization, annual landings ranged from 250 to 800 t from 1999 onwards. Between 2013 and 2024, there was a shift in the landings, with an increase in Helgeland, Nordland County (65°–66° N), and a decrease in the Trøndelag area due to the implementation of new diving restrictions and reorganization of the fishery in the area. By 2024, the catch north of 65° N accounted for approximately 50% of the total harvest of 412 t. The reported landings north of 65° N have remained stable since 2012, confirming the presence of a harvestable population in the northern area. Already in 1937, Bodø (~67° N) was recognized as the northernmost distribution of the great scallop (Dons 1937). During a monitoring survey conducted by the Norwegian Institute of Marine Research (IMR) in 2001, the early findings of Dons (1937) was confirmed, where the northernmost recording of a small population of great scallops was done by scientific divers at Grønholmen, west of Bodø (67°16′ N, 14°09′ E; Ø. Strand, pers. comm.).

In the early 1950s, Soot‐Ryen (1951) reported a record of a fresh‐looking *P. maximus* valve from the north‐eastern coast of Andøy (69° N, 16° E), and a few nonverified observations of the great scallop north of Vesterålen are reported in Artsdatabanken (Norwegian national biodiversity database). These are limited to single valves, except from a record of a single live *P. maximus* done during maerl bed mapping in 2016 at Storflatholmen, Harstad Municipality, Troms and Finnmark County (69° N, 16° E) (documented by photo and verified by E. Grefsrud). However, no recordings of scallop populations have been reported in these areas.

The great scallop can reach an age of > 20 years (Mason 1983) and in Norwegian waters, the scallop is typically found partly buried in coarse to fine shell sand at depths ranging from 5 to 60 m, with the highest concentration occurring at 15–30 m depth (Wiborg and Bøhle 1974; Strand and Parsons 2006). The great scallop prefers relatively warm water (> 10°C) and high salinity (> 30) (Beaumont and Budd 1982; Strand et al. 1993; Strand and Brynjeldsen 2003), and temperature is considered the primary factor limiting the distribution range (Chauvaud et al. 1998; Laing 2000; Shephard et al. 2010). After spawning and fertilization, scallop larvae remain pelagic for approximately 15–38 days at temperatures ranging from 15°C to 18°C (Comely 1972; Gruffydd and Beaumont 1972; Le Pennec et al. 2003; Magnesen et al. 2006). However, *P. maximus* larvae have been found to develop and survive at temperatures as low as 10°C–12°C. At lower temperatures (4°C–7°C), larvae and postlarvae experience slower growth rates and lower survival rates compared to juveniles and adults (Beaumont and Budd 1982). During the juvenile stage, Strand and Brynjeldsen (2003) observed 100% mortality at 2°C and 30% mortality at 4°C–5°C, whereas Strand et al. (1993) demonstrated that scallops with a shell height of 30 mm, held at 5°C in western Norway, experienced minor mortality (10%–15%) after 4 weeks.

The hydrographical conditions reflect to a high degree the distribution pattern of *P. maximus* in Norwegian waters. The environment encompasses temperate to subpolar conditions, with a strong influence of Atlantic water, particularly from 60° to 65° N (Sætre and Aure 2007). The coastal current, primarily driven by the outflow from the Baltic and freshwater discharge from land, flows northwards with a typical speed of 0.2–0.5 m/s (Sætre and Aure 2007). Outside the coastal current, the North Atlantic currents flow northwards with a mean average transport of 4.2 Sverdrup (Orvik et al. 2001). This water mass is saline with relatively high temperatures. Consequently, the water masses along the Norwegian coast are relatively warm compared to the latitudes. The annual temperature span is greatest in southern Norway, close to the surface, and decreases northwards. The southernmost populations of the great scallop in Norway are scattered and occur relatively deep, primarily at depths of 25–50 m and are believed to be limited by the hydrographical conditions mainly due to influx of cold brackish water from the Baltic during late winter and early spring (Strand and Parsons 2006). While summer temperatures are highest in the south and decrease northwards, winter temperatures reach their peak around 60°–65° N due to the strong influence of Atlantic water in this region (Figure 1, Sætre and Aure 2007). Winter temperatures decrease both to the south and north of this area, and the lowest temperatures close to the surface are found in February/March. The highest scallop abundances are observed in Mid and Northern Norway (63°–66° N; Wiborg and Bøhle 1974, Strand and Vølstad 1997). The occurrence of relatively large populations (~5–6 individuals per square meter) of great scallops in these areas coincides with the presence of warm Atlantic water masses near the coastline (Sætre and Aure 2007; Strand and Parsons 2006). North of these high‐abundance areas, the temperature decreases to what is expected to be the lower limit for *P. maximus* , with winter temperatures below 4°C during prolonged periods.

As one moves from the coast into the fjords, the topography generally becomes steeper, and the upper water column experiences greater seasonal oscillations in temperature and salinity. Due to the combined effects of topography and environmental oscillations, the outer coastal area is perceived as suitable for great scallop populations.

The dispersion and distribution pattern of sedentary species, such as *P. maximus* , is predominantly controlled by the transport during the larval phase. Therefore, the distribution of great scallops in Norway can be linked to the hydrodynamical properties of the water masses along the coast. Given these circumstances, the Norwegian coastline serves as an intriguing region for studying the biological responses and changing habitats resulting from altered environmental conditions. In this paper, we present our observations on scallop populations in northern Norway related to historical environmental data and previously documented environmental preferences of the species. Our aim is to evaluate whether the presence of warmer water masses along the coast is likely to explain the northward expansion of *P. maximus* along the Norwegian coast over the past 20 years.

## 2 Material and Methods

### 2.1 Great Scallops, Mapping, and Monitoring

As part of the “National marine habitat mapping program” (2007–2019), we utilized a vessel‐towed underwater camera platform to map the occurrences of the great scallop ( *Pecten maximus* ) in Norwegian coastal areas. The mapping effort was moving successively from south toward north. Real‐time video footage was collected along survey tracks to document the presence of scallops. The selection of survey track positions was based on a combination of monitoring data from the Institute of Marine Research, expert knowledge, topographic information, shell sand distribution obtained from sea maps (utilizing the Directorate of Fisheries map tool Yggdrasil), scallop fishery landing data, and information provided by local divers.

**FIGURE 2:**
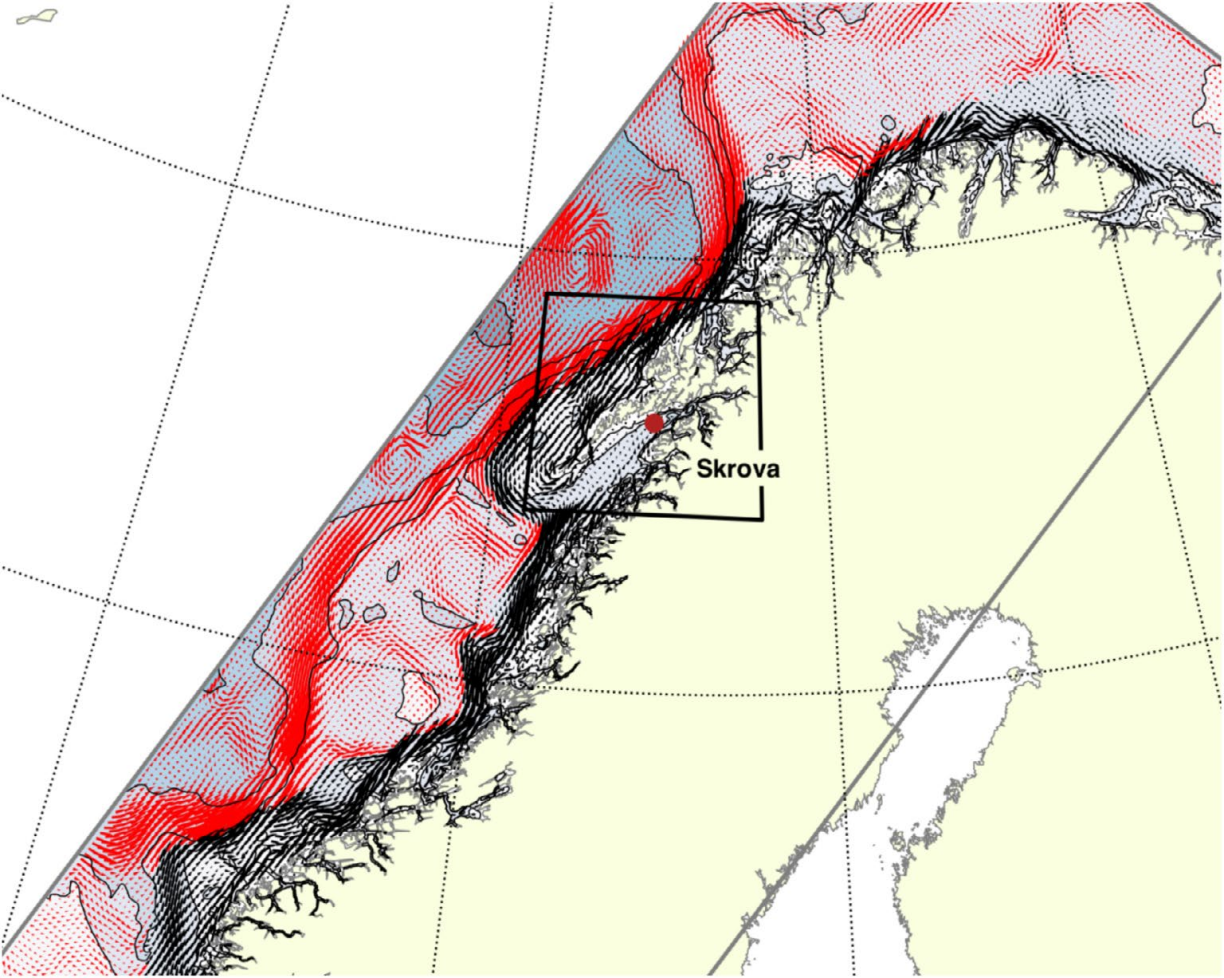
The Norwegian coastline with the modeled coastal current marked with black arrows and Atlantic current in red. The model domain is boxed in gray, and the area of interest for the northerly distribution of great scallop ( *Pecten maximus* ) is framed in black. The coastal station Skrova where temperature and salinity are observed is marked with a red dot.

During the period between 2011 and 2015, we mapped the area ranging from 65° to 69° N (Figure 2). Three hundred and twenty‐seven unique stations were covered in this effort, with track length ranging from 37 to 1762 m (average track length 404 m). Assuming that each meter tracked equals 1 m^2^, about 132 km^2^ sea bottom were covered during the surveys. In total, approximately 6500 live scallops were observed. By analyzing the number of observed scallops in the video recordings and considering the track length, we estimated the approximate abundance of scallops per unit area where scallops occurred (scallops per square meter).

**FIGURE 3:**
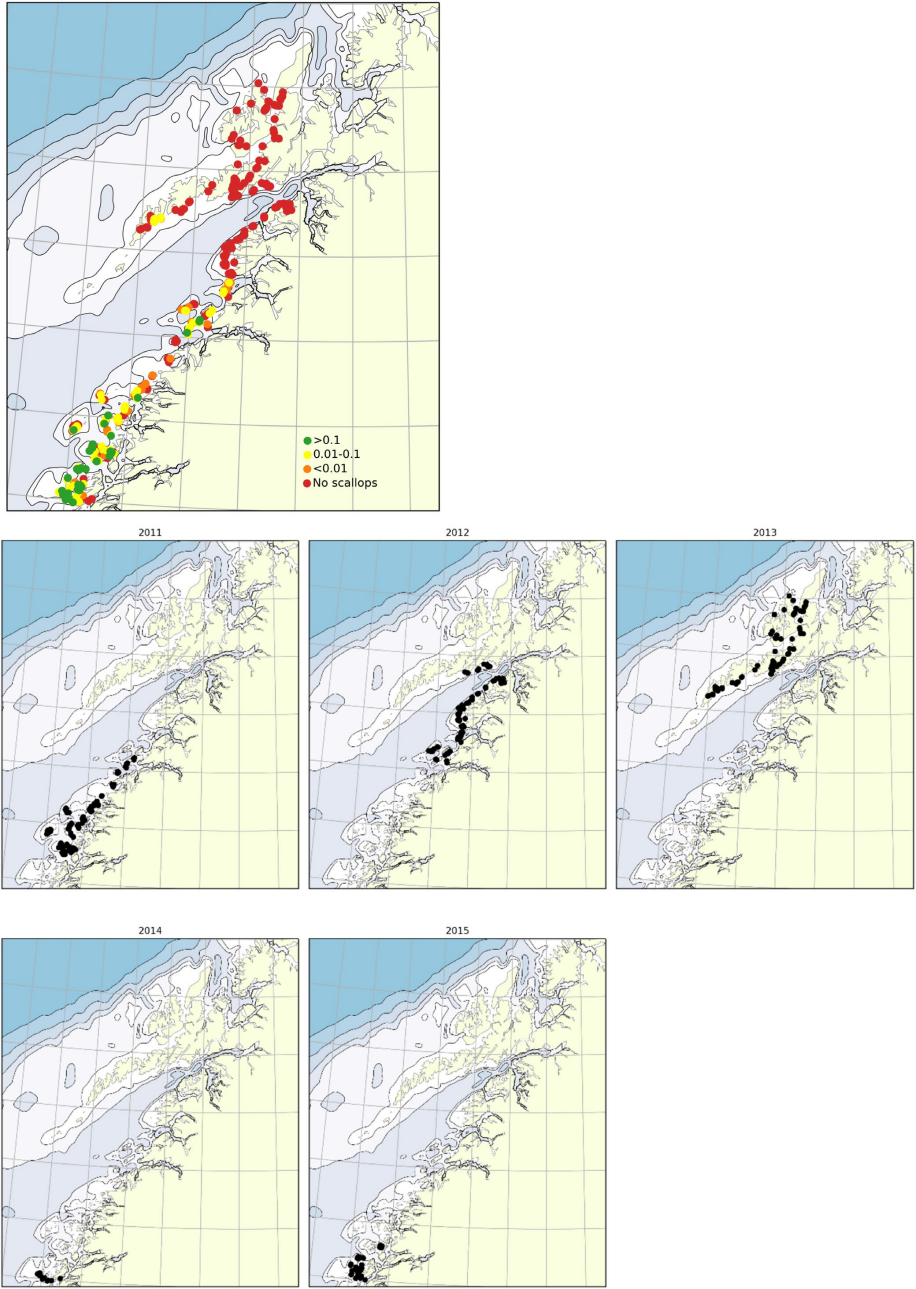
Observed occurrence (*N* scallops per meter) of the great scallop ( *Pecten maximus* ) in the period 2011–2015 (video survey) and 2017 (scuba diving), categorized as follows: no observations in red, < 0.01 in orange, 0.01 to < 0.1 in yellow, and > = 0.1 in green (upper figure). The timing of the observations from 2011–2015 shown in lower figure.

In 2017, we conducted revisits to 27 locations (67.5°–68° N) to confirm the previous registrations made in 2012 and 2013. These revisits involved underwater video at all locations, and scuba diving was conducted to collect specimens for further analysis at the five locations where scallops were observed (Nusfjord, Skjelfjord, Litle Kvaløya, Enokskjæret, and Værholmen; Figure 3). We measured the scallop shell height of collected scallops using vernier calipers, and scallop age was determined by carefully examining the growth rings present on the flat valve of the scallop shell, following the method described by Mason (1957) and ICES (2020).

### 2.2 Environmental Data

To evaluate the hydrodynamic conditions in relation to the mapped scallop populations in the northern part of the distribution area, we utilized observed temperature and salinity data from the coastal station Skrova (68° N; Figure 2, Institute of Marine Research 2023), along with results from a hydrodynamic model. Hydrography measurements at the coastal station have been obtained since 1940. Hydrography observations are conducted about twice a month, but based on previous research projects, the hydrography observations are intensified during early spring in some years. To remove false trends due to uneven sampling between the years, the hydrography data was fitted. The data were linearly interpolated to 14‐day intervals using the griddata function from the Python package scipy.interpolate to reduce errors in the dataset caused by irregular and modified sampling routines.

To gather information on the spatio‐temporal areas that could potentially serve as suitable habitat for scallops, the observational dataset was supplemented with results from the Regional Ocean Model System (ROMS, http://myroms.org, Albretsen 2011). The model was configured with a horizontal resolution of 800 × 800 m and 35 sigma layers in the vertical. It incorporates fresh water supply, external forcing at the surface, and outer ocean conditions from model fields, as described in Albretsen (2011) and Albretsen et al. (2022). The model has been demonstrated to realistically replicate the temperature and salinity patterns along the coast (Asplin et al. 2020). Additionally, the fjord circulation is well captured 87% of the time according to Dalsøren et al. (2020). The numerical model was run in hindcast mode, providing hourly fields of temperature, salinity, and currents from 1995 to 2021, for the entire coastline.

### 2.3 Criteria Used to Map Potential Habitats From Models

The geographical area where *P. maximus* potentially may settle and grow to adult reproductive stages was estimated based upon the environmental conditions. The environmental criteria *P. maximus* needs to settle and survive to adult reproductive stages were based on scientific literature. *P. maximus* are found at depths down to 60 m, with the highest concentrations between 15 and 30 m depth (Wiborg and Bøhle 1974; Strand and Parsons 2006).

Temperature is considered the primary factor limiting the distribution range of great scallop (Laing 2000; Chauvaud et al. 1998; Shephard et al. 2010). The great scallop's temperature tolerance varies with the different life stages, where the larvae and postlarvae are more sensitive to low temperatures compared to juveniles and adults (Beaumont and Budd 1982; Strand et al. 1993; Strand and Brynjeldsen 2003). Increased mortality and reduced growth rates are observed at 4°–5°C (Beaumont and Budd 1982; Strand and Brynjeldsen 2003). Also, the spawning of great scallops is dependent on temperature and is observed to occur when the temperature exceeds 7°–9°C (observed at 64° N; Strand and Nylund 1991).

As for temperature, the great scallops' tolerance to low salinity events is shown to be different through the scallops' developmental stages and varies with temperature (Davenport et al. 1975). Hatchery‐reared spat showed increased mortality and reduced growth at salinity below 28–30 (Laing 2002) and combined with reduced temperature (from 18°C to 15°C) scallop spat (1.4–2.3 mm shell height) held at salinities of 20 and 25 showed increased mortality, reduced growth, and reduced byssal attachment compared to those held at 30 (Christophersen and Strand 2003). Further, mortality of juveniles exceeded 50% at salinities below 26 at low temperatures (5°C; Strand et al. 1993).

Subsequently, the geographical areas that met the following environmental criteria were delimited:

- Depth less than 60 m.
- Consecutive period of less than 14 days with seabed temperature below 4°C.
- Seabed temperature reaching a maximum value above 7°C.
- Seabed salinity exceeding a minimum of 28.

These criteria were used to identify and define potential suitable habitat for the great scallop based on the environmental information extracted from the hydrodynamical model.

## 3 Results

### 3.1 Great Scallops, Mapping, and Monitoring

During the marine habitat mapping surveys conducted from 2011 to 2015, populations of the great scallop ( *Pecten maximus* ) were documented using an underwater video camera within the latitude range of 66°–68.1° N (Figure 3). Generally, the populations exhibited a decrease in density and an increase in distance between occurrences as one moved further north. South of Bodø, populations of great scallops with a density exceeding 0.1 scallops per square meter were observed (Figure 3; green dots) up to 67.4° N. Beyond this point, great scallops were found at lower densities (< 0.01 scallops per square meter, Figure 3; yellow dots) and in the range of 0.01–0.1 scallops per square meter (Figure 3; orange dots) up to 67.6° N. North of 67.6° N, the only recorded occurrence of great scallops was at 68° N on the western side of Vestfjorden, with low densities (< 0.01 scallops per square meter). The northernmost verified live population of great scallops was reported in Nusfjord (68.1° N, 13.3° E), where a small but dense population (0.01–0.1 scallops per square meter) was found (Grefsrud et al. 2015).

**FIGURE 4:**
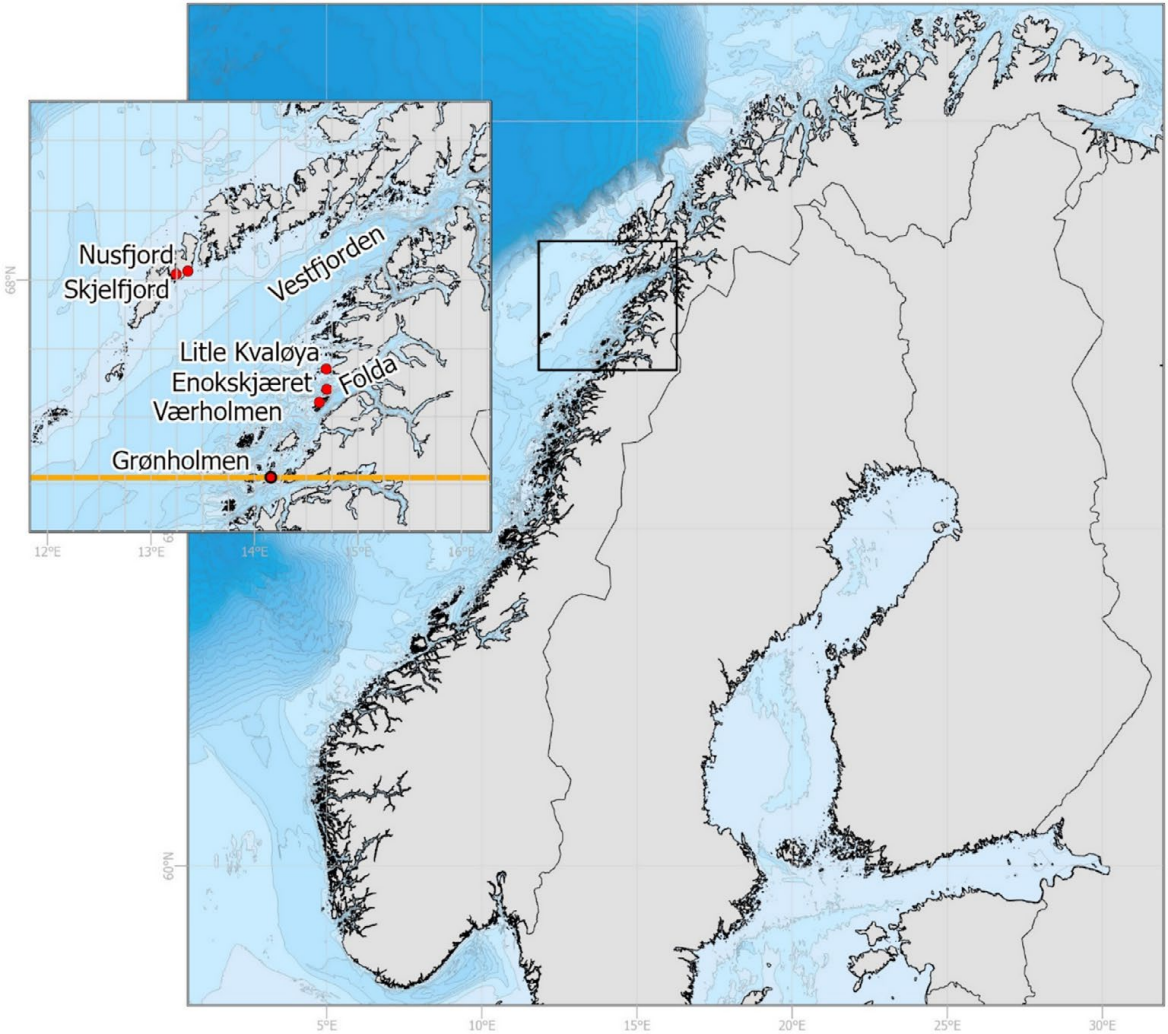
Map showing the Norwegian coast and the location of the five stations surveyed by scuba diving in 2017. The orange line with the red circle represents the northernmost distribution range of the great scallop *Pecten maximus* in 2001.

**FIGURE 5:**
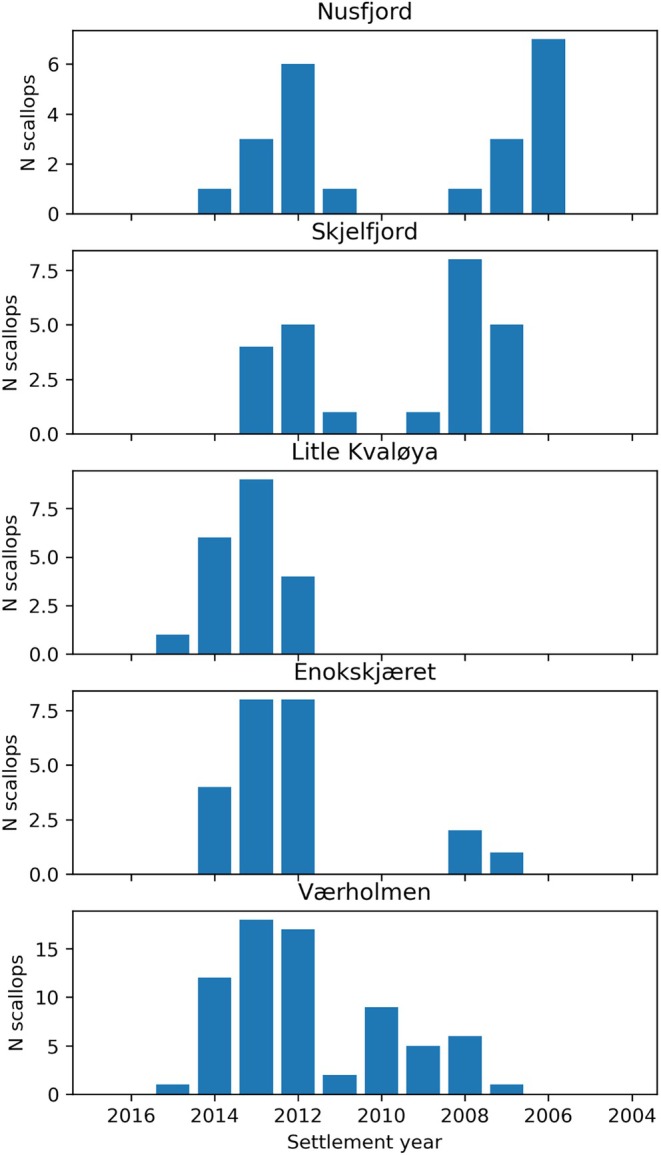
Age distribution of great scallops ( *Pecten maximus* ) collected by scuba diving at five different survey stations in 2017.

During a revisit to the northern populations in 2017, 27 stations were covered using drop camera. At five of the stations, live *P. maximus* was observed and samples were collected by scientific SCUBA diving: Værholmen (*n* = 71), Enokskjæret (*n* = 23), Litle Kvaløya (*n* = 20), Nusfjord (*n* = 23), and Skjelfjord (*n* = 25) (Figures 4 and 5).

Scallop age was determined to be between 2 and 11 years; the oldest scallops were from the 2006 year class and the youngest from the 2015 year class (Figure 5). The age distribution indicated relatively stable recruitment patterns since the mid‐2000s at the southernmost station (Værholmen) and the two northernmost stations (Nusfjord and Skjelfjord), the recruitment pattern seemed to be more unstable at Enokskjæret, where few recruits were exhibited in 2007–2008, followed by annual recruitment since 2012. Furthermore, divers in 2017 did not find any scallops older than 5 years at Litle Kvaløya, while in 2012, no scallops were observed in this area.

### 3.2 Environmental Data

**FIGURE 6:**
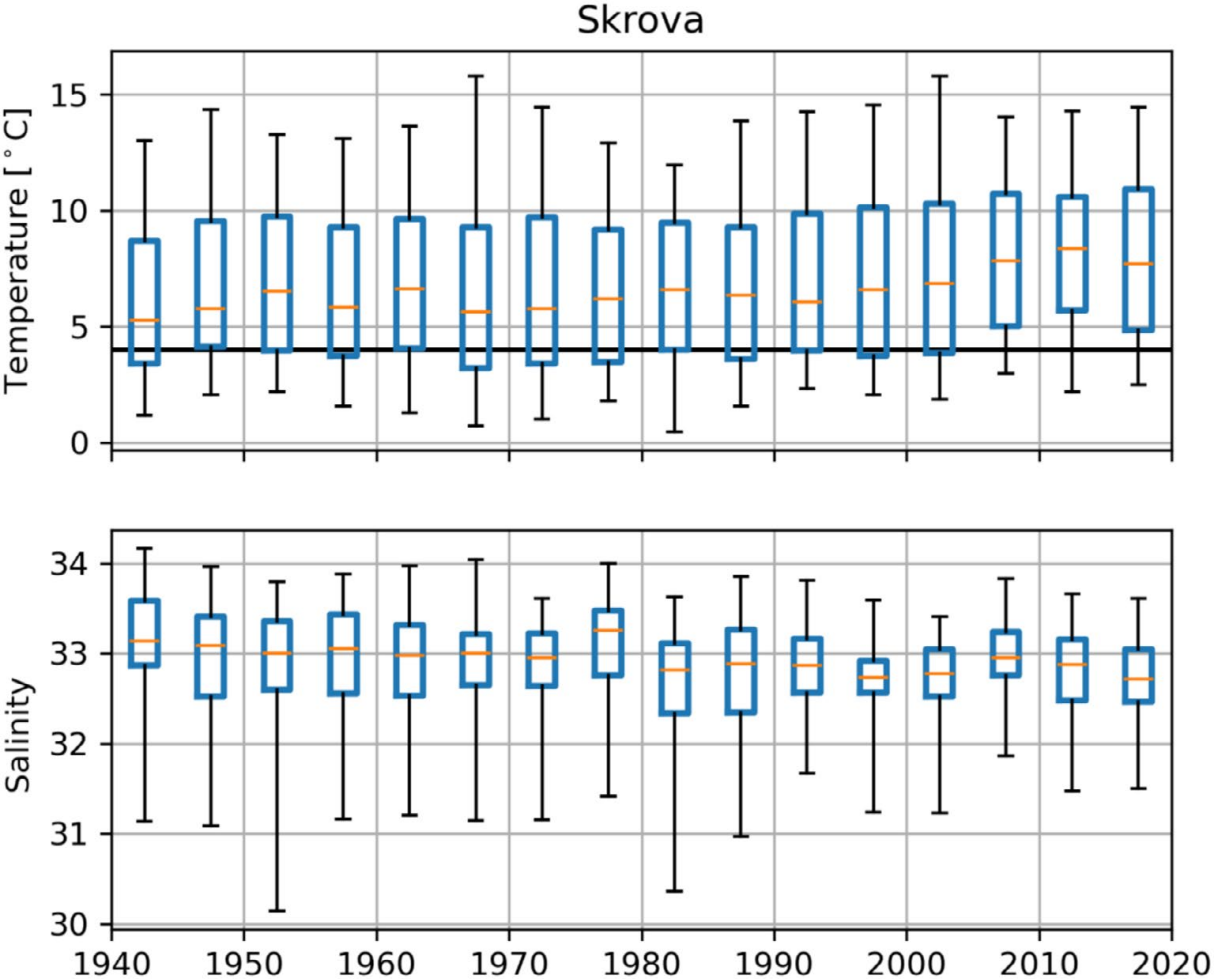
Observed temperature and salinity at 10 m depth from the coastal stations Skrova (68.1° N). The orange line marks the median value, the blue box shows the 25‐ to 75‐percentiles, the black lines mark the minimum and maximum value grouped in 5‐year intervals. Horizontal lines mark the assumed lower tolerance for great scallop ( *Pecten maximus* ).

**FIGURE 7:**
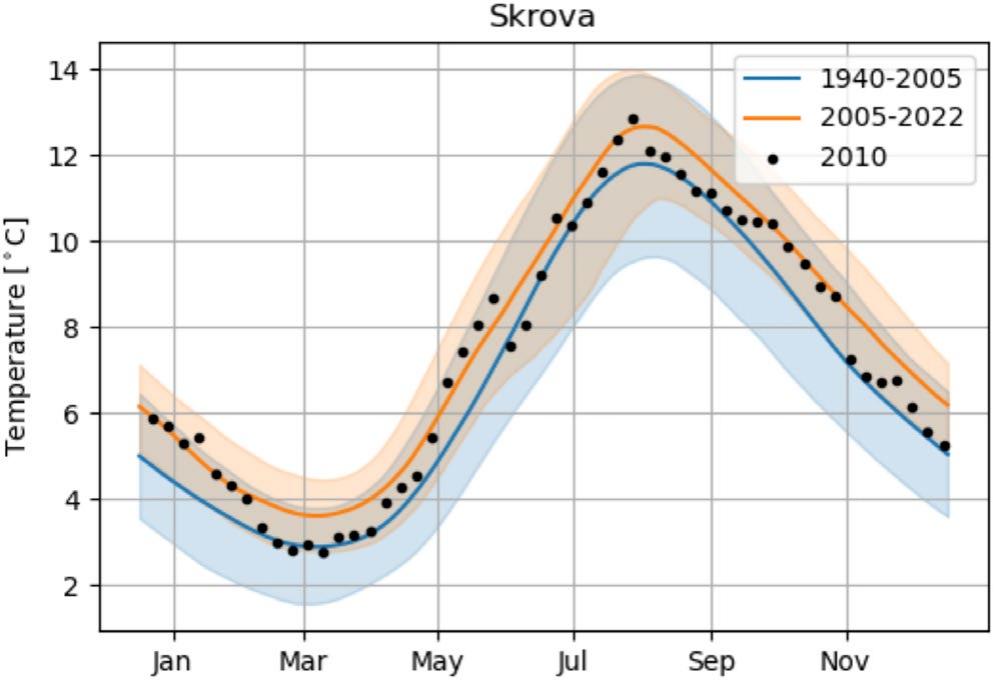
Observed temperature from Skrova (68.1° N) at 10 m depth normalized from 1940 to 2005 (blue) and 2005 to 2022 (orange). The line shows the median temperature, the shaded area shows the 5–95 percentiles. Black dots show the observed temperature during 2010.

The observed temperature at a depth of 10 m from the coastal station Skrova displayed seasonal fluctuations ranging from approximately 3° to 13°C (Figure 6). Over the period from 1940 to 2022, there was an overall increase in temperature of about 1°C, with a shift occurring around 2005 (Figure 6). When comparing the periods of 1940–2005 and 2005–2022, the average winter minimum temperature increased from 3°C to 4°C, and from 12°C to 13°C during summer (Figure 7). Despite the temperature increase, winter temperatures below 4°C continued to be observed every year, although the duration of such low temperatures has decreased. Prior to 2005, these temperatures were typically observed for three consecutive months during winter (Figure 7). However, after 2005, the timespan of winter temperatures below 4°C reduced to 1 month. Notably, the winter temperature in 2010 was relatively low compared to the observations from 2005 to 2022, as it approached the mean temperature during the 1940–2005 period.

In terms of salinity, measurements at Skrova indicated seasonal fluctuations between 30 and 34 at a depth of 10 m, with lower salinity during the summer. Unlike temperature, no apparent alteration in salinity around 2005 was observed (Figure 6).

**FIGURE 8:**
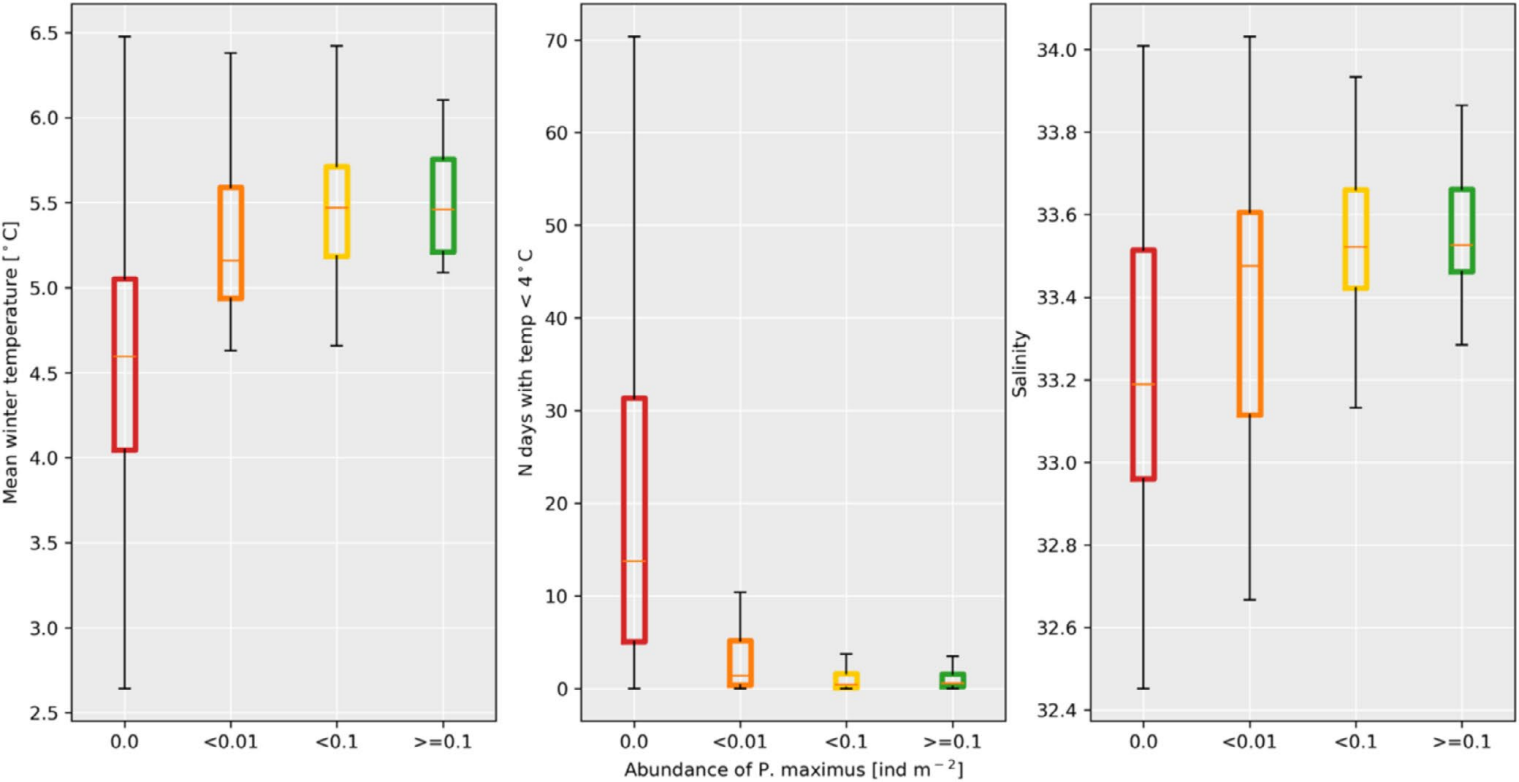
Modeled environmental parameters mean 2005–2017 for locations grouped by the observed abundance of the great scallop ( *Pecten maximus* ). Mean temperature during March (the coldest month, left panel), the average number of days with temperatures < 4°C (mid panel), and the salinity minimum (right panel). The orange line marks the median value, the box shows the 25‐ to 75‐percentiles, the black vertical lines mark the most extreme, nonoutlier value.

The observed density of *P. maximus* increased with increasing winter temperature and increasing salinity minimum at the observed location. Correspondingly, the observed density decreased with increasing number of days with temperature below 4°C (Figure 8). The strongest relationship was found for mean winter temperature, where the winter temperature was found to be significantly higher at the 146 stations where scallops were observed (5.4°C, ±0.4), in comparison to the 182 stations where no observations were made (4.6°C, ±0.8), *t*‐value = 11.8 (*p* < 0.001, calculated using scipy.stats.ttest_ind in Python). Furthermore, the number of days with temperatures below 4°C was lower at the stations where great scallops were present (2.1 days, ± 3.5) compared to the stations without scallop observations (21.3 days, ±21.0), *t*‐value = 10.8 (*p* < 0.01). While no significant difference was found for the annually averaged salinity comparing stations with and without great scallops, it was observed that the stations without great scallops had lower annual minimum salinity (33.5, ±0.3) compared to the stations where great scallops were found (33.2, ±0.4) *t*‐value = 8.2 (*p* < 0.001). Note, the mean salinity both for stations with and without *P. maximum* were well within the assumed preferred salinity conditions (> 28).

**FIGURE 9:**
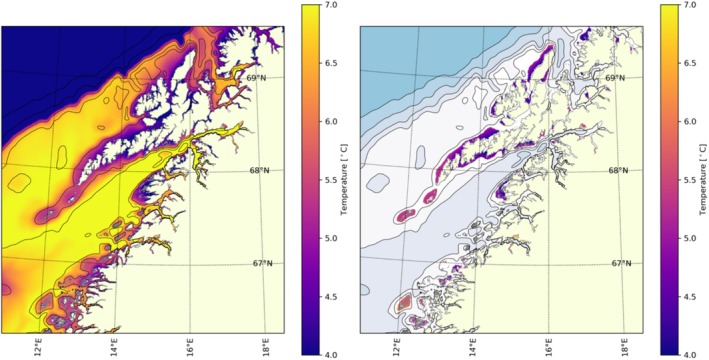
Modeled mean seabed temperature in March 2005–2017 (left side). Modeled mean seabed temperature in March in areas with depth < 60 m, minimum salinity > 28, and less than 14 days consecutive with temperature < 4°C during 2005–2017 (right side).

During winter, heat loss to the atmosphere resulted in lower seabed temperatures in shallow areas compared to greater depths (Figure 9, left side). The mean temperature in March, which is the coldest month, for the areas that met the criteria mentioned in Section 2.3 (indicating the areas where the criteria for depth and salinity set for *P. maximus* were fulfilled; Section 2.3), is displayed in Figure 9 (right side). Within the fjords, the topography tends to be steep, and there are limited shallow areas suitable for potential scallop habitats. Additionally, the winter temperature and summer salinity within the fjords were lower compared to those along the coastline. The seabed area north of 65° N, which was shallower than 60 m in depth and had an average winter temperature exceeding 4°C, covered the highlighted area in Figure 9, right side.

## 4 Discussion

Poleward shifts in marine species have been reported for both fish and shellfish, which can have significant impacts on ecosystem services, fisheries, and the communities reliant on them for food security (Hollowed et al. 2013; Kortsch et al. 2015; Fossheim et al. 2015). The expansion of sedentary species with limited mobility during the adult stages depends on factors such as larval advection, suitable settlement habitat, and an environment that supports growth, survival, and maturation in adulthood (MacLeod 1985).

Considering the typical northward flow of the Norwegian coastal current, which has an average speed of 0.2–0.5 m/s, with variability on the same scale, there is the potential for sedentary species to extend their distribution toward northern regions during the pelagic larval stages as reported for faster moving boreal fish (Kortsch et al. 2015, Fossheim et al. 2015). Historical observations of live specimens and fresh‐looking valves found at 66°–69° N in the 1930s and 1950s suggest that great scallops have been transported into the Lofoten area and that some individuals have successfully settled and survived there in the past (Dons 1937; Soot‐Ryen 1951). However, until 2001, the northernmost viable *P. maximus* population recorded during monitoring surveys, was at Grønholmen, west of Bodø (67°16′ N, 14°09′ E). Revisiting the area in 2011/2012, well established scallop populations were observed north to Enokskjæret (67°36′ N, 14°41′ E), suggesting that the great scallop had moved further north during the last decade. Since no scallops were found north of Folda in 2012, we suggest that this could be a result of the local hydrodynamic conditions. The circulation patterns in Vestfjorden are characterized by the presence of eddies, meanders, and fronts, which exhibit variability depending on wind conditions (Mitchelson‐Jacob and Sundby 2001). Additionally, the fjords on the eastern side of Vestfjorden are known for their outflow of brackish water, with 90% of the freshwater in Vestfjorden originating from these fjords. This outflow of brackish water creates barriers (Mitchelson‐Jacob and Sundby 2001; Myksvoll et al. 2014; Johnsen et al. 2016). We hypothesize that these outflows from the fjords can potentially impede the establishment of great scallop populations through advection, hindering the transport of great scallop larvae across the outflow from the fjords, for example, from south to north of the Folda outlet. When returning in 2017, a scallop population consisting of individuals < 5 years age, was recorded at Litle Kvaløya north of Folda, showing that the scallop larvae had crossed the hydrodynamic barrier, probably around 2012. The sporadic recruitment pattern found at Enokskjæret however, still suggests that the local conditions some years may be suboptimal for *P. maximus* resulting in empty year classes. The same recruitment pattern has been observed in the Western part of Norway, where years with high freshwater input combined with low winter temperatures have resulted in empty year classes (Strand 1986).

The variable circulation pattern resulting from eddies, meanders, and fronts may help explain why scallop recruitment in Vestfjorden did not occur consistently in a south–north or outer–inner gradient. No scallops were found northward along the coastline from Litle Kvaløya, and westward along the southern coastline of Lofoten except the two small but seemingly viable populations in Nusfjord and Skjelfjorden. Once transported into an area, the postlarvae of great scallops require suitable substrate for settlement and survival during their later stages. They thrive in subtidal waters and on substrates such as shell sand. Generally, there are more suitable areas for scallop settlement in the mid and outer parts of the coast, as the fjords are characterized by steep topography that may not be as conducive for scallop habitat. Although the sediment type was not specifically studied in the present research, the Geological Survey of Norway has conducted mapping of shell sand as part of the “National Marine Habitat Mapping Program” in the area. The mapping revealed that most of the shallow areas in the region have suitable sediment conditions for great scallops. Therefore, the substrate type is not considered a major limiting factor for the northerly distribution of great scallops in this area.

The great scallop was observed in higher abundance in areas with slightly higher salinity minimum; however, the salinity range observed in most of the coastal area remained well above the assumed tolerance limit of around 28 (Davenport et al. 1975; Strand et al. 1993). Along the Norwegian coast, higher salinity values typically indicate a higher proportion of warm Atlantic water in the coastal regions. Increasing salinity is therefore often accompanied by increasing temperatures and may explain why scallops were more abundant in areas with higher salinity. Also, the food content for the great scallops may be different in the two water masses; however, for this study, we did not have information about the food availability for scallops in the Atlantic nor the coastal water. This should be looked further into in future studies.

Although we cannot know for sure, our observations strongly indicate that the small but viable populations of *P. maximus* found in the Lofoten Islands had not been established for a long period of time. Age distribution (2–11 years) and lack of empty shells that tend to accumulate in areas with scallop populations support this assumption. Also, it is less likely that scallop populations would be established without it being noticed by the scientific community or local divers. Recreational diving is very common in Norway, and ahead of the mapping survey in 2011, local divers and diving clubs were contacted to get more information about scallop occurrences in the survey area. Only one local diver confirmed that she had observed *P. maximus* in the Lofoten area (Nusfjord). Her first observation was from 2006, and since then she had only observed a few live specimens in the area. Although observations from a single diver cannot be used as scientific evidence alone, these observations combined with the mapping results, age determination, and historical environmental data support our assumption that the great scallop only recently has established viable populations in the Lofoten area, indicating that the great scallop, like many other warm‐water species, is moving northwards along the Norwegian coast, mainly explained by the increasing water temperatures (Brattegard 2011; Hollowed et al. 2013; Kortsch et al. 2015; Fossheim et al. 2015; Kjesbu et al. 2022).

Both observations and models have consistently demonstrated that the temperatures in the study area have been sufficiently high for great scallop spawning and survival during spring and summer each year for several decades. However, the winter temperatures have been low enough for where the mortality of great scallops is observed to increase (< 4°C). Our findings show that scallops were primarily observed in areas with warmer water during the winter and in regions with fewer days of temperatures below 4°C. While there is no definitive lower threshold for what adult *P. maximus* can tolerate, the early life stages appear to be more susceptible than the later stages. Although juvenile and adult great scallops can endure longer periods of temperatures as low as 4°C–5°C (Beaumont and Budd 1982; Strand and Brynjeldsen 2003), physiological and behavioral changes have been documented, along with an increased vulnerability to predation by sea stars and crabs (Strand et al. 1993, 2004). The relationship between temperature and the occurrence of great scallops was evident and statistically significant. Analysis of the coastal observational data revealed that while these low temperatures typically persisted for consecutive months between 1940 and 2005, the duration decreased to approximately 1 month after 2005. By utilizing results from a hydrodynamical model, we discovered that the environmental conditions necessary for the growth and survival of great scallops to the adult stages were present in extensive areas both north and south of the Lofoten Islands.

Despite the overall increasing trend in water temperature since 2005, it is important to note that cold winters can still occur. In 2010, for instance, the temperature was observed to be close to the normal range recorded between 1940 and 2005 during winter. Interestingly, this particular year class was only observed at one of the five stations analyzed, specifically at Værholmen station, which is the southernmost location included in this study. The future trajectory of coastal water temperature is largely uncertain. The temperature along the Norwegian coast is known to undergo long‐term oscillations in addition to the climatic alterations (Albretsen et al. 2012; Kjesbu et al. 2022), and climate predictions specifically downscaled to coastal and fjord scales are limited. Additionally, there are indications that water transport and genetic flow along the coastline are reduced north of the Lofoten Islands where the coastal current divides into two branches (Sætre and Aure 2007; Ribeiro et al. 2022). As a result, estimating the further expansion of great scallops into northern areas becomes challenging. However, future efforts focusing on habitat modeling, incorporating the data presented here and potentially considering information on larval dispersion, could provide valuable insights.

## 5 Conclusion

The great scallop populations in Norway have historically had a northernmost distribution limited to approximately 66°–67° N (Dons 1937; Soot‐Ryen 1951; Strand, personal observation). However, with the observed 1°C increase in temperature over the past two decades, the great scallop has shown movement further north, establishing two small but viable populations as far as 68° N.

Indeed, studies have reported on the polar shift of habitat due to climate change, including in the context of marine species (Hollowed et al. 2013; Kortsch et al. 2015; Fossheim et al. 2015). In the case of great scallops along the Norwegian coastline, the increasing temperatures since 2005 and the documented recruitment at the northernmost stations during the same period suggest that winter temperature historically acted as a limiting factor for their survival in this area.

By considering the data from scallop mapping and diving surveys along the Norwegian coast, it can be concluded that the distribution area of *P. maximus* has expanded northwards. This shift in distribution probably began in the early 2000s, and since neither the occurrence of suitable habitat nor low salinity seems to be limiting factors, the temperature increase, with reduction of mean winter temperatures and periods with low (< 4°C) from weeks to a few days, stands as the most plausible explanation for the northwardly expansion. Stuart‐Smith et al. (2017) conclude in a meta‐analysis that seasonal temperature range rather than latitude or ocean currents seems to be the most critical factor for understanding species distribution limits, especially in temperate species such as *P. maximus* . This conclusion as well as our results aligns well with the broader trend of polar shifts in species distributions observed in response to climate change. In a recent climate scenario study, Gallagher and Albano (2023), in addition to showing that *P. maximus* distribution would expand northwards, their results also indicated an increase in abundance of the great scallop. The authors suggest that such changes in distribution and abundance may increase the harvest potential of this species. Since 2013, a new commercial fishery has been established in Nordland County in Norway, supporting this prediction. However, knowledge about these northernmost populations size, recruitment, and resilience to increased fishing pressure is still limited.

The ongoing shifts in species distributions and the potential expansion of range boundaries provide valuable insights into the ecological consequences of climate change. Continued research and monitoring efforts in this area will be crucial to our understanding of how marine ecosystems are adapting and evolving in the face of these environmental changes, and further this knowledge will contribute to future advice on sustainable use and conservation of marine resources and ecosystem services.

## Author Contributions

**Ingrid A. Johnsen:** conceptualization (equal), data curation (equal), formal analysis (equal), investigation (equal), methodology (equal), validation (equal), visualization (equal), writing – original draft (equal), writing – review and editing (equal). **Ellen Sofie Grefsrud:** conceptualization (equal), data curation (equal), formal analysis (equal), investigation (equal), methodology (equal), project administration (equal), validation (equal), visualization (equal), writing – original draft (equal), writing – review and editing (equal).

## Conflicts of Interest

The authors declare no conflicts of interest.

## Supporting information


Appendix S1

Appendix S2

## Data Availability

The environmental data are already publicly available. Monitoring/mapping and biological data on *Pecten maximus* is submitted with the manuscript as Excel files.
